# Supplementary material for: Identification of mobile development issues using semantic topic modeling of Stack Overflow posts
Source: PeerJ Comput Sci. 2023 Oct 24;9:e1658. doi: 10.7717/peerj-cs.1658 (PMC10703021; doi:10.7717/peerj-cs.1658)
Supplement: Supplemental Information 2 [file peerj-cs-09-1658-s002.docx]

**Table A1.** List of keywords used to identify mobile-related posts

| **Hardware** | **Platform** | **Development** |
| --- | --- | --- |
| iphone | android | react-native |
| ipad | ios | ionic |
| huawei | mobile | cordova |
| galaxy | tizen | nativescript |
| tablet | wear-os | phonegap |
| nexus | windows-phone | titanium |
| nokia | blackberry | cocoa-touch |
|  | webos | coronasdk |
|  | palm | renderscript |
|  | symbian | java-me |
|  | garnet-os | qt-mobility |
|  | bada | xamarin |
|  | meego | objective-c |
|  | maemo | swift |
|  |  | flutter |
|  |  | kotlin |
|  |  | xcode |
|  |  | appcelerator |
|  |  | sencha |
|  |  | rubymotion |
