# Supplementary material for: Identification of mobile development issues using semantic topic modeling of Stack Overflow posts
Source: PeerJ Comput Sci. 2023 Oct 24;9:e1658. doi: 10.7717/peerj-cs.1658 (PMC10703021; doi:10.7717/peerj-cs.1658)
Supplement: Supplemental Information 3 [file peerj-cs-09-1658-s003.docx]

**Table A2.** List of tags used to identify and extract mobile-related posts

| **ID** | **Tag** | **TRT** | **TST** | **ID** | **Tag** | **TRT** | **TST** |
| --- | --- | --- | --- | --- | --- | --- | --- |
| 1 | android | 91.88 | 100.00 | 34 | uicollectionview | 78.08 | 0.80 |
| 2 | ios | 84.90 | 48.78 | 35 | bluetooth | 65.46 | 0.79 |
| 3 | swift | 59.00 | 13.38 | 36 | parse-platform | 54.71 | 0.79 |
| 4 | objective-c | 56.96 | 12.15 | 37 | xamarin.ios | 66.74 | 0.78 |
| 5 | iphone | 66.02 | 10.67 | 38 | android-ndk | 80.76 | 0.78 |
| 6 | xcode | 53.50 | 6.02 | 39 | swift3 | 57.96 | 0.78 |
| 7 | flutter | 65.40 | 5.74 | 40 | android-gradle-plugin | 86.68 | 0.78 |
| 8 | react-native | 54.20 | 4.93 | 41 | opengl-es | 76.27 | 0.78 |
| 9 | android-studio | 75.71 | 4.67 | 42 | android-viewpager | 96.47 | 0.76 |
| 10 | android-layout | 97.39 | 4.15 | 43 | android-emulator | 82.70 | 0.71 |
| 11 | uitableview | 70.95 | 3.35 | 44 | autolayout | 79.45 | 0.70 |
| 12 | android-fragments | 96.67 | 3.18 | 45 | google-play | 88.04 | 0.69 |
| 13 | kotlin | 60.40 | 3.13 | 46 | bitmap | 59.89 | 0.68 |
| 14 | listview | 67.12 | 2.54 | 47 | notifications | 59.88 | 0.67 |
| 15 | cordova | 53.14 | 2.39 | 48 | imageview | 91.72 | 0.66 |
| 16 | android-intent | 96.70 | 2.16 | 49 | broadcastreceiver | 97.99 | 0.65 |
| 17 | xamarin | 60.82 | 2.14 | 50 | android-actionbar | 97.72 | 0.63 |
| 18 | android-activity | 96.03 | 2.02 | 51 | uiscrollview | 69.43 | 0.63 |
| 19 | android-recyclerview | 94.94 | 1.96 | 52 | storyboard | 65.92 | 0.59 |
| 20 | ionic-framework | 53.81 | 1.91 | 53 | sprite-kit | 60.07 | 0.58 |
| 21 | ipad | 54.66 | 1.39 | 54 | camera | 54.11 | 0.56 |
| 22 | cocoa-touch | 54.42 | 1.38 | 55 | uibutton | 67.39 | 0.55 |
| 23 | mobile | 56.90 | 1.20 | 56 | admob | 76.49 | 0.55 |
| 24 | webview | 76.44 | 1.20 | 57 | fragment | 93.56 | 0.55 |
| 25 | core-data | 57.71 | 1.20 | 58 | uinavigationcontroller | 65.74 | 0.54 |
| 26 | android-listview | 97.26 | 1.11 | 59 | android-sqlite | 89.67 | 0.53 |
| 27 | android-asynctask | 97.52 | 1.04 | 60 | retrofit | 89.36 | 0.52 |
| 28 | textview | 96.55 | 0.98 | 61 | uikit | 69.80 | 0.52 |
| 29 | uiview | 69.47 | 0.94 | 62 | sharedpreferences | 90.07 | 0.52 |
| 30 | xamarin.android | 67.82 | 0.88 | 63 | uiwebview | 65.76 | 0.51 |
| 31 | push-notification | 63.85 | 0.86 | 64 | in-app-purchase | 77.47 | 0.51 |
| 32 | android-edittext | 97.36 | 0.85 | 65 | uiimageview | 65.14 | 0.50 |
| 33 | uiviewcontroller | 70.53 | 0.83 | 66 | apk | 87.91 | 0.50 |
