# Supplementary figures and images for: Identification of mobile development issues using semantic topic modeling of Stack Overflow posts
Source: PeerJ Comput Sci. 2023 Oct 24;9:e1658. doi: 10.7717/peerj-cs.1658 (PMC10703021; doi:10.7717/peerj-cs.1658)

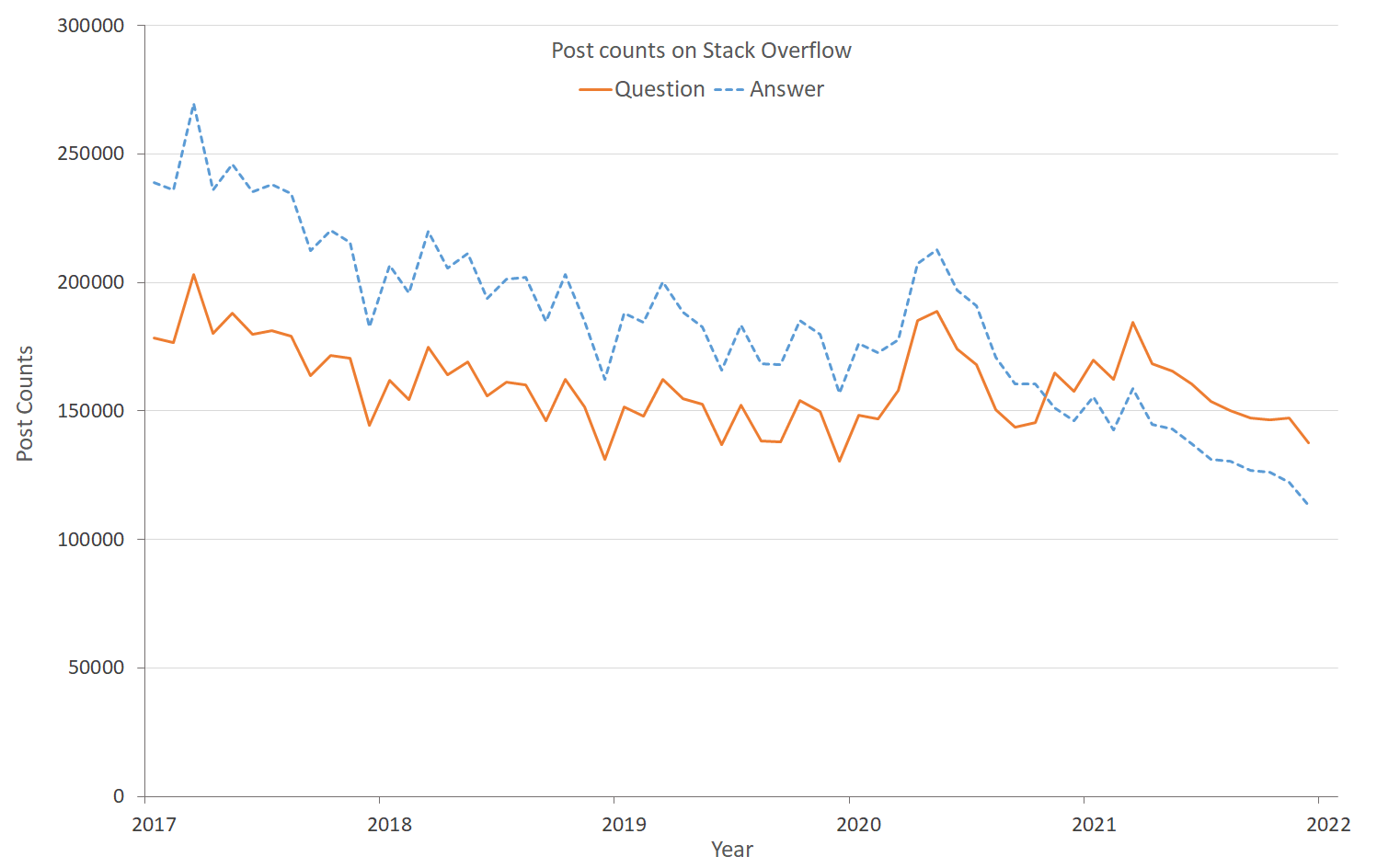

Supplement: Supplemental Information 4 [file peerj-cs-09-1658-s004.png]

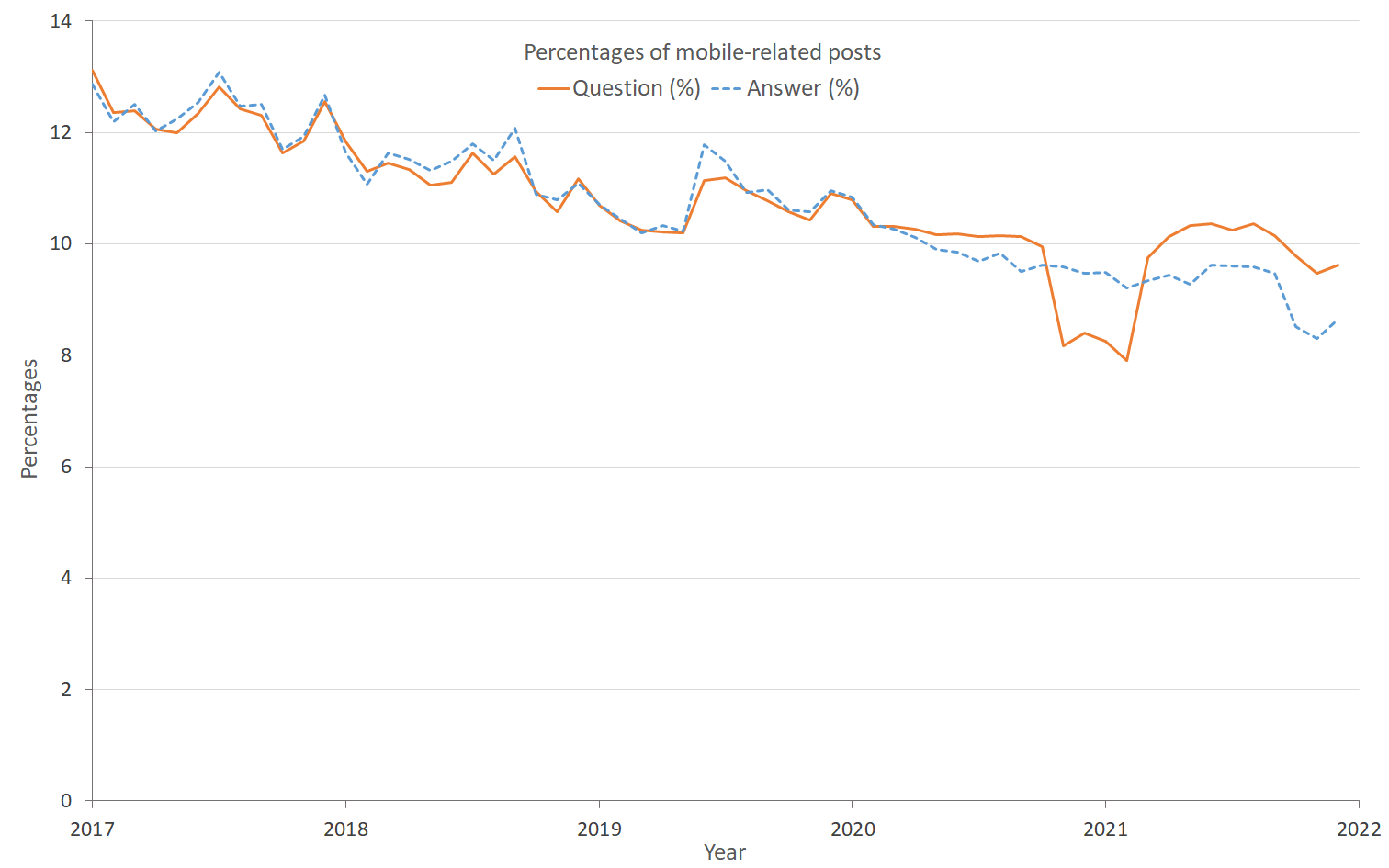

Supplement: Supplemental Information 5 [file peerj-cs-09-1658-s005.png]

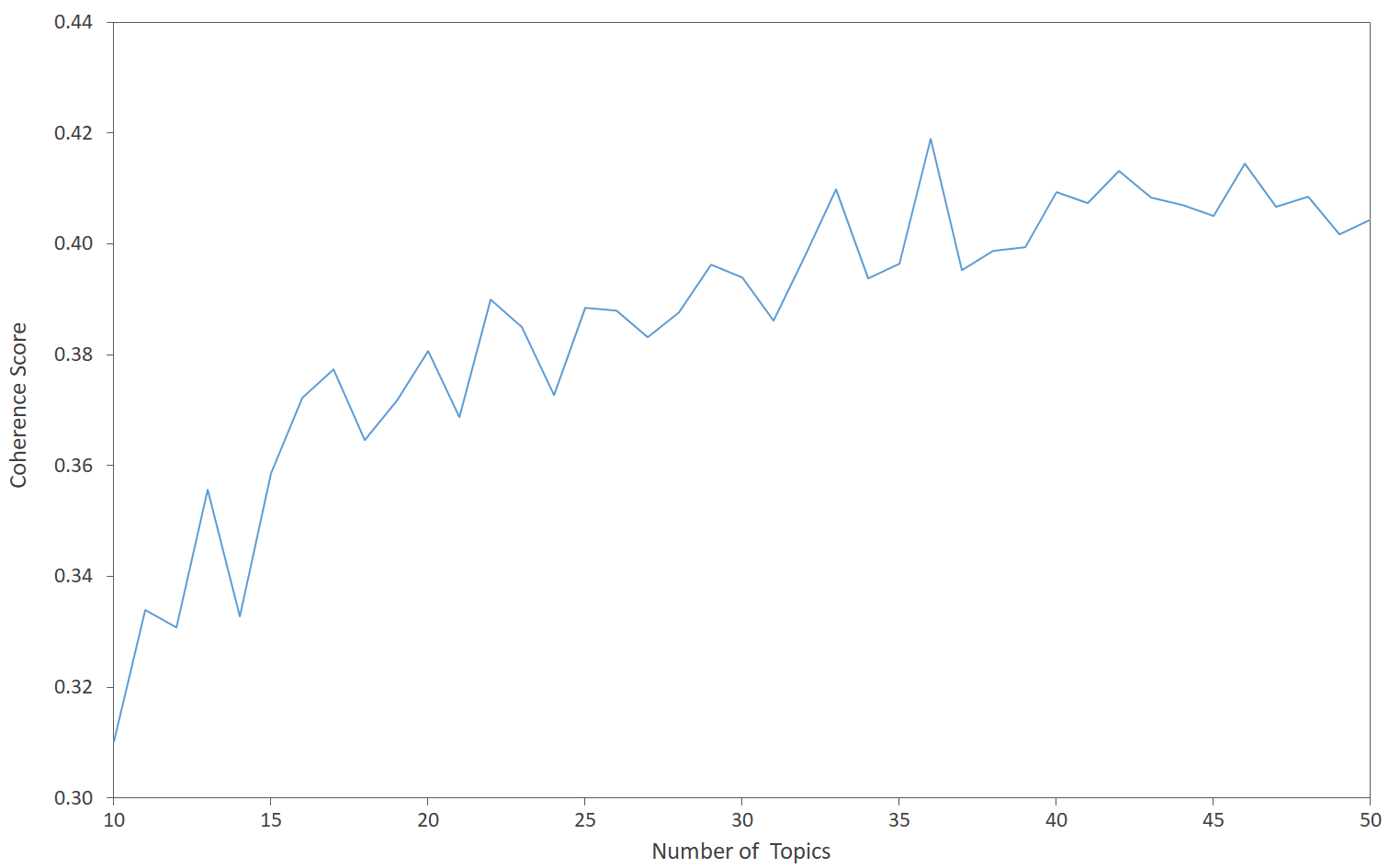

Supplement: Supplemental Information 6 [file peerj-cs-09-1658-s006.png]
